# The Progress of Pharmacy and Pharmaceutical Chemistry

**Published:** 1905-06

**Authors:** Oliver C. M. Davis

**Affiliations:** Lecturer in Chemistry and Materia Medica at University College, Bristol


### THE PROGRESS OF PHARMACY AND PHARMACEUTICAL CHEMISTRY.

BY

OLIVER C. M. DAVIS, B.Sc. Lond.,

*Lecturer in Chemistry and Materia Medica at University College, Bristol.*

As every issue of the *Bristol Medico-Chirurgical Journal* contains reports on medical and surgical cases, thus bringing before its readers the great advances which have been made and are still being made in medicine and surgery, it seems not unlikely that members of the medical profession may be interested in reading a short article bringing forward a few instances to illustrate the progress of pharmacy and pharmaceutical chemistry.

In giving an outline of some of the more important changes which have taken place in pharmaceutical methods it is first necessary to consider the origin of the practice of pharmacy. During the early Egyptian period the study of medicine and alchemy lay in the hands of the priests, and was a "holy" art. Many Greek writers, such as Plato and Herodotus, gained the confidence of these practitioners, and were consequently enabled to penetrate their secrets; but it was not until the latter half of the fifteenth century that Basil Valentine inaugurated a new era by his attempt to apply chemical preparations in medicine. Up to that period alchemy, the study of the transmutation of metals, slowly accumulating a mass of knowledge invaluable to science, had held the field. Basil Valentine, by his daring introduction of chemical substances, commenced a new era called the Iatro-Chemical, and Paracelsus, who said "the object of the chemist is to prepare medicine, not to make gold," in the first half of the sixteenth century broke new ground for chemistry and medicine by uniting them both. Paracelsus practised that which he preached, for he enriched medicine with a large number of valuable preparations. By this means a tremendous impetus was given to the higher development of the apothecary's calling; for before the time of Paracelsus apothecaries' shops were nothing more than stores for roots, herbs, syrups, and confections of every kind, the preparation of the last-named being carried out exclusively in them.

The making of new medicines presupposed an acquaintance with chemical facts and processes, so that the pharmacists had to be continually striving to attain to this knowledge, and pharmacy in the proper sense of the word came into being at this point. Drug-shops, in which artificial preparations then began to be made, were the nurseries of energetic chemists, who, especially in the succeeding generation, played an important part in the building-up of the scientific system.

The work done with organic substances also led to their practical application in medicine and in daily life, and also to improvements in the mode of preparation.

Speculations have been made regarding the knowledge of the remedial properties of natural substances. Probably all plants and plant-products were originally tried as food, the trials resulting in the discovery of that which we now call therapeutic action. From time immemorial man has undoubtedly placed a high value upon the medicinal qualities of plants. Egyptian formulæ for preparations from plants have been found dating as far back as 2,700 B.C.

Apparently the first pharmacopœia sanctioned by civil authority was published at Nürnberg in 1545, being chiefly founded on the practice of Galen. In this book are to be found certain preparations closely resembling many in the present British pharmacopœia. It also includes such substances as granite, sapphire, clay, and dried goat's blood, the efficacy of which may be open to doubt. A pharmacopœia published at Augsburg, in 1646, by the authority of the Town Council, contains many good recipes, and opens with a dissertation to the pharmacist on his duty towards his neighbour.

In 1618 was published the first edition of the *Pharmacopœia Londinensis*, in which numerous simples are mentioned, including one interesting item, "Cranium humanum violente morte extinctum."

Many works of that period throw an interesting light upon the nature of the preparations with which the pharmacies of those old apothecaries were stored. Some of the herbs and simples that they kept are in as good repute as ever, but the names of others bring a smile, especially when the purposes to which they were put are known. For instance, Burton in his wonderful collection of the world's specifics for melancholy (*Anatomy of Melancholy*, 1621) sets down, among the medicaments to be taken inwardly to "alter" melancholy, "wines, as of hellebore, bugloss, hops, epithyme, endive, succory," &c. His "consisting" medicines are "conserves of violets, maidenhair, borage, bugloss, roses," &c. Confections: "Treacle, mithridate, eclegmes, or tinctures." For outward application are suggested "oils of camomile, violets, roses, &c., ointments, alablastritum, populeum, &c., liniments, plasters, cerates, cataplasms, frontals, epithymes, sacks, bags, odoraments, posies," &c. Doubtful cures for melancholy, but a fair sample of the preparations of the age.

The first British pharmacopœia was published in 1864; it was a digest of the works previously issued from London, Edinburgh, and Dublin. It is a small-sized manual, and might conveniently be carried in the pocket. The chemical formulæ therein mentioned are worthy of special notice, that for water being HO. In many cases the dualistic formulæ of Berzelius are used, copper sulphate being expressed as $CuO.SO_3+6HO$.

Directions for applying chemical tests to pharmaceutical products are mentioned, and volumetric solutions of certain reagents are also included. A conspicuous feature is the absence of any stated doses, an omission which at the present day, with countless preparations in use, would be a serious matter for all concerned.

The edition published in 1885 is a great improvement on former issues. One marked difference is the introduction of quantitative tests in connection with the more powerful substances; it insists, for example, that powdered opium shall contain not less than 9.5 nor more than 10.5 per cent. of morphine, and describes a method for determination.

The standardisation of extract of nux vomica is also included, as also are improved methods for the detection of impurities and adulterations in various preparations.

The latest edition, published in 1898, is in many respects far ahead of any of its predecessors. The number of preparations requiring standardisation is greatly increased, and the methods of standardising are also improved. As an example, in the 1885 issue extract of nux vomica is adjusted to contain a definite percentage of total alkaloids (strychnine and brucine). As the former substance is far more toxic than the latter, a method of separation has been devised, depending on the formation of insoluble strychnine ferrocyanide, the corresponding brucine compound being soluble. The 1898 extract, therefore, contains a definite proportion of strychnine.

Some two years ago a method of separating the two alkaloids strychnine and quinine was introduced by Harrison and Gair. It depends on the greater solubility of strychnine tartrate in solution of Rochelle salt than of quinine tartrate. Since preparations containing both alkaloids are largely used, it is a great advantage to be able to determine the percentage of each present.

Although in many cases a chemical investigation is of great service in determining the value of certain drugs, yet in other cases this method of examination leaves much to be desired. In such instances a physiological test is the great criterion, and it is now possible to procure a number of preparations whose potency have been determined physiologically and more satisfactorily than with the aid of chemical reagents.

An important advance in medical practice is the increased use of the isolated active principles of plants, instead of such preparations as extracts and tinctures. These isolated principles are especially valuable where exact dosage, combined with small volume, are essential, as in the preparation of hypodermic injections.

Cocain, ergotinine, digitalin, hyoscyamine, eserine, strophanthin, atropine, and numerous other alkaloids and glucosides possessing marked therapeutic properties can now be obtained in a high state of purity.

In a similar manner bodies of animal origin are now isolated and purified for medicinal use. To this class belong some of the ferments, and also such substances as "takamine."

In the preparation of the commoner chemicals electrical methods have been coming into vogue of recent years. Phosphorus, which was formerly made by a lengthy process involving the initial preparation of phosphoric acid, is now prepared by heating a mixture of calcium phosphate and finely crushed coke in an electric furnace. Potassium chlorate is also produced, especially in Switzerland and Sweden, where water power is available for the generation of electricity, by electrolysis of potassium chloride.

The manufacture of innumerable galenicals in compressed form has come into practice in late years, and much is to be said both for and against such products. It is a great convenience for the physician to have at his disposal preparations containing invariable amounts of medicament in a convenient form for administration, but there is a danger in the possibility of indiscriminate use of certain of these products by the general public in cases where their action may be undesirable and even dangerous.

The development of bacteriology has led to the introduction of many new pharmaceutical products. The great bacteriologist, Pasteur, and the eminent surgeon, Lord Lister, have revolutionised modern surgery, the result being that there is a huge demand for antiseptic dressings of all kinds, for the manufacture of which the wholesale chemist is called upon. In the preparation of everyday pharmaceutical products, such as hypodermic injections, eye lotions, &c , the water used is usually sterilised by boiling immediately before use. Another notable advance in pharmacy is the increasing use as therapeutic agents of the serum of animals immunised by bacteria.

Perhaps the greatest advance made in the province of pharmaceutical chemistry since the Iatro-chemical period is the introduction of large numbers of synthetic compounds, the preparation of which often involves many complicated reactions and a thorough knowledge of organic chemistry. Within the last few years several hundred of these compounds have been introduced for medicinal use. The synthesis of such substances may be regarded as dating from Liebreich's investigation of the action of chloral in 1868, based on Liebig's discovery of chloroform in 1831.

The synthetic compounds come chiefly under the head of narcotics, such as sulphonal ; antipyretics, such as phenacetin ; and antiseptics, such as salol. The knowledge of whether a substance is decomposed or not in passing through the body, and, if decomposed, a knowledge of the decomposition products and their therapeutic effect, was regarded by Liebreich as fundamental in pharmacological investigations. Salol, to take an example of an intestinal disinfectant, passes through the stomach unchanged, but in the duodenum is decomposed by the pancreatic juice into salicylic acid and phenol; that is, phenol is set free where its action is required.

Betol similarly gives rise in the intestine to salicylic acid and B. naphthol, a powerful antiseptic, but less toxic than phenol. A large amount of work has been carried out on these lines resulting in the preparation of many valuable products.

In conclusion, a few cases may be cited illustrating the interdependence of chemical constitution and physiological activity.

The problem of determining the constitution of many complex vegetable substances is at present occupying the attention of many eminent chemists, and important results have already been obtained. For example, the codeine molecule differs from the morphine molecule by one methyl ($CH_3$) group, and yet the actions of these alkaloids differ considerably. Aconitine, the powerfully poisonous alkaloid extracted from aconite root, differs from the non-toxic alkaloid aconine by one benzoyl ($C_6H_5CO$) and one acetyl ($CH_3CO$) group. Brucine differs from strychnine by two methoxy ($CH_3O$) groups, and this may account for its markedly diminished toxic action.

Similarly, the molecules of quinine and cinchonine differ only by one methoxy group.

Following these observations, substitution products of certain alkaloids have been placed on the market, among which may be mentioned "heroin" (diacetyl-morphine), "peronine" (the hydrochloride of benzoyl morphine), and "dionine" (the hydrochloride of mono-ethyl-morphine).

After observing the effects of introducing various groups into compounds of known physiological re-action, similar groups have been introduced into synthetic compounds, a description of which would be beyond the scope of the present paper. To this class belong such bodies as phenacetin, sulphonal, trional, tetronal, &c.

The great importance of this fascinating branch of investigation which is still in its infancy must be evident, and the solution of the problem of therapeutic action, as a result of the internal structure of the molecule, should in the future give us a series of drugs of graduated physiological activity, and do away with many of the empirical methods of to-day, and tend to reduce pharmacology to an exact science.

